# Dosimetric effect of silicone‐based gel on skin surface during volumetric modulated arc therapy for breast cancer

**DOI:** 10.1002/acm2.70070

**Published:** 2025-03-13

**Authors:** Tenyoh Suzuki, Shingo Ohira, Mami Ogita, Takeshi Ohta, Yuki Nozawa, Masanari Minamitani, Takuya Hayashi, Shigeki Saegusa, Toshikazu Imae, Tsuyoshi Ueyama, Atsuto Katano, Hideomi Yamashita, Weishan Chang, Keiichi Nakagawa

**Affiliations:** ^1^ Department of Radiological Sciences, Graduate School of Human Health Sciences Tokyo Metropolitan University Arakawa‐ku Tokyo Japan; ^2^ Department of Comprehensive Radiation Oncology, Graduate School of Medicine The University of Tokyo Bunkyo‐ku Tokyo Japan; ^3^ Department of Radiology The University of Tokyo Hospital Bunkyo‐ku Tokyo Japan

**Keywords:** StrataXRT, silicone‐based gel, skin surface dose, volumetric modulated arc therapy, breast cancer

## Abstract

**Purpose:**

This study aims to quantify and compare the dosimetric effects of varying thicknesses of StrataXRT, a silicone‐based gel, and other topical agents on the skin surface during volumetric modulated arc therapy (VMAT) for breast cancer.

**Methods:**

A VMAT plan was created for breast cancer treatment using a female RANDO phantom with a prescription dose of 50 Gy in 25 fractions. The planning target volume (PTV) encompassed the left breast and the regional lymph nodes. Irradiation was performed using a 6 MV photon beam. Three topical agents (StrataXRT, Hirudoid Soft Ointment, and RINDERON‐Vs Ointment) having eight thicknesses (0.0–1.5 mm) were evaluated. Dosimetry was conducted using Gafchromic EBT4 films at three anatomical locations—subclavicular, medial, and lateral aspects of the left breast.

**Results:**

Mean dose enhancement ratios (DERs) of 102%–116% were observed in VMAT for topical agent thicknesses of 0.1–0.5 mm, increasing to 116%–126% at 1.0 and 1.5 mm. Among the evaluated agents, StrataXRT consistently exhibited the lowest DER, with a statistically significant difference (*p* < 0.05).

**Conclusions:**

This study demonstrated that StrataXRT exhibited the lowest DER among the evaluated topical agents in VMAT for breast cancer. Thicknesses exceeding 0.5 mm potentially exceeded the threshold dose for acute skin reactions.

## 1 INTRODUCTION

Breast cancer is the most common malignant tumor among women worldwide.^1^ Treatment options for breast cancer include surgery, pharmacotherapy, and radiotherapy. Among these, radiotherapy plays an important role in breast cancer treatment as an adjuvant treatment after surgery, reducing mortality rates and the risk of breast cancer recurrence.^2^, ^3^, ^4^ In pathologically‐confirmed lymph node metastasis with an elevated risk of recurrence, adding regional lymph nodes in the irradiation field is recommended. volumetric modulated arc therapy (VMAT) offers a more uniform dose to the breast and regional lymph nodes than conventional radiotherapy.^5^, ^6^ However, radiation dermatitis is the most prevalent adverse event in radiotherapy for breast cancer, affecting approximately 95% of patients. This condition, characterized by pruritus, and pain, impairs the patient's quality of life.^7^, ^8^

In clinical practice, topical agents, primarily corticosteroid‐based ointments, are recommended to prevent radiation dermatitis.^9^, ^10^ While corticosteroids have demonstrated efficacy in alleviating radiation dermatitis,^11^ prolonged use may lead to adverse effects, such as cutaneous atrophy, telangiectasia, and an increased susceptibility to infections.^12^, ^13^ In recent years, StrataXRT, a silicone‐based gel, has been developed to prevent and treat radiation dermatitis and has been shown to significantly reduce the risk of severe (grade 3) radiation dermatitis in patients undergoing radiotherapy for breast cancer compared with standard care.^14^ Upon drying, StrataXRT forms a thin, flexible, protective layer that is, gas‐permeable and waterproof, promoting a moist wound‐healing environment. While StrataXRT is expected to provide therapeutic effects without inducing the adverse reactions associated with corticosteroids, its optimal efficacy requires continuous application 24 h a day, 7 days a week.

The use of topical agents before radiotherapy remains a subject of debate among researchers due to concerns regarding the bolus effect and electron scattering.^15^, ^16^ Studies on conventional irradiation techniques have demonstrated that topical agents with thicknesses of 0.5 and 1.0 mm increased the surface doses to 130%–135% and 197%–238%, respectively compared with that of a thickness of 0.0 mm.^17^, ^18^ However, removing these agents before treatment may cause friction‐related skin damage. Previous research on increased surface doses during radiotherapy with topical agents in situ primarily focused on ointments and creams, utilizing single‐beam perpendicular irradiation.^15^, ^17^, ^19^ Considering the unique properties of StrataXRT and the complexity of VMAT, it is crucial to evaluate the effect of the former on skin surface dose in the specific context of breast cancer radiotherapy.^20^, ^21^

This study aims to quantify and compare the dosimetric effects of varying thicknesses of StrataXRT and other topical agents on skin surfaces in VMAT for breast cancer. To determine whether StrataXRT offers dosimetric advantages over conventional treatments, we included other topical agents in our experimental design for direct comparison.

## 2 METHODS

### 2.1 Topical agents

Three types of topical agents were used in this study (Table 1): StrataXRT (Stratpharma, Basel, Switzerland), a silicone‐based gel; Hirudoid Soft Ointment (Maruho, Osaka, Japan), a non‐steroidal agent; and RINDERON‐Vs Ointment (Shionogi Pharma, Osaka, Japan), a corticosteroid‐based agent. These agents were selected based on our institutional clinical practice, representing different categories of topical treatments used for radiation dermatitis management. The AUX‐220 analytical balance (Shimadzu, Kyoto, Japan) was utilized to measure the mass of 1.0 cm^3^ for each topical agent to determine the mass density, and the measurement was repeated three times. The mass of each topical agent was subsequently measured to obtain eight different thicknesses (0.0, 0.1, 0.2, 0.3, 0.4, 0.5, 1.0, and 1.5 mm), and the measured topical agents were enclosed using reclosable poly bags with a thickness of 0.04 mm.

### 2.2 Radiochromic film dosimetry

Surface dose measurements were conducted using Gafchromic EBT4 radiotherapy films (LOT. 05012303; Ashland, NJ, USA). The films were placed between 10 and 11 cm‐thick Tough Water Phantoms (Kyoto Kagaku, Kyoto, Japan) and irradiated in a 10 cm × 10 cm field at a source‐surface distance (SSD) of 90 cm using a 6 MV photon beam from a Versa HD linear accelerator (linac) system (Elekta, Stockholm, Sweden). Nine films were prepared, namely one unexposed film and eight films exposed to doses of 25, 50, 100, 150, 200, 300, 400, and 500 monitor units (MU). To determine the absorbed dose to the corresponding MU, we conducted dose measurements three times using a Farmer Ionization Chamber 30013 (PTW, Freiburg, Germany), delivering 100 MU each time, and the other irradiation conditions were the same as those of the films.

All films were scanned 24 h after irradiation using a GT‐X980 flatbed scanner (Seiko Epson, Nagano, Japan) in the transmission mode with a resolution of 72 dots per inch, 48‐bit color depth, and portrait orientation, with all color collections turned off. To minimize the lateral response artifact effect, we positioned the films at the center of the scanning area. The calibration curve was generated by plotting the measured analog‐digital converter (ADC) values against the calculated absorbed doses and fitting the data with a third‐degree polynomial function using the least‐squares method using DD‐Analysis software (v.12.2; R‐tech, Nagano, Japan).

The dose enhancement ratio (DER) is the ratio of the measured dose in the presence of a topical agent to the dose obtained in the absence of a topical agent. To ensure measurement accuracy, we focused on the DER rather than absolute surface doses, effectively eliminating the influence of measurement uncertainties and dose enhancement effects attributed to electron contamination.

### 2.3 Conventional irradiation

**FIGURE 1:**
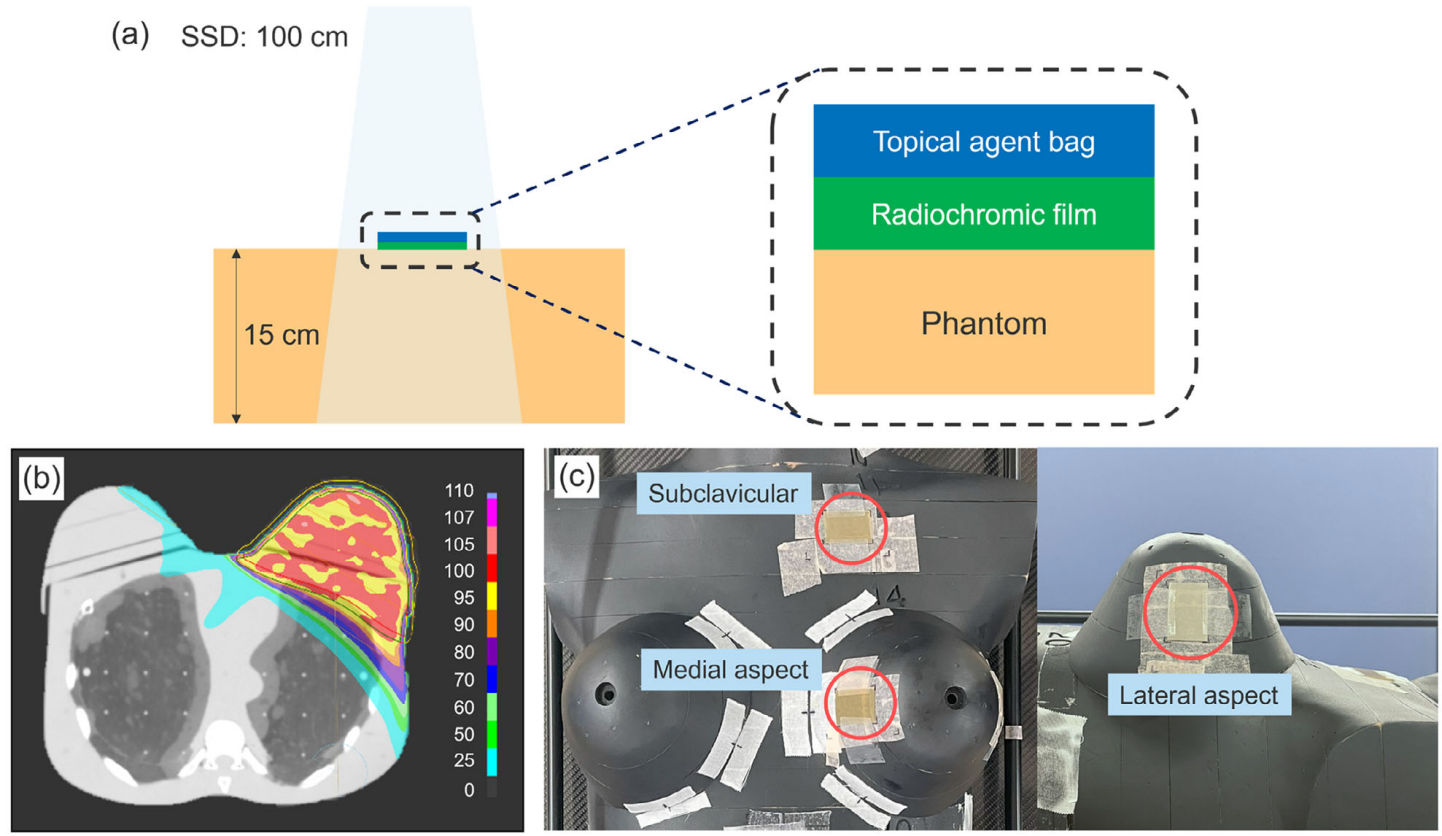
Experimental setup for conventional and VMAT irradiation, and dose distribution for VMAT irradiation. VMAT, volumetric modulated arc therapy.

In this study, a series of experiments was conducted under simplified conditions before conducting experiments under complex conditions. Figure 1(a) shows the experimental setup used for conventional irradiation measurements. A 20 mm × 30 mm film was placed on top of a 15 cm‐thick Tough Water Phantom at its center, and a reclosable polybag containing a topical agent was positioned above the film. The gantry, collimator, and couch angles were maintained at 0°, and the topical agents were irradiated with 200 MU in a 10 cm × 10 cm field at an SSD of 100 cm using a 6 MV photon beam from a Versa HD linac.

### 2.4 VMAT irradiation

A female RANDO phantom (The Phantom Laboratory, NY, USA) was scanned using an Aquilion LB computed tomography (CT) scanner (Canon, Tokyo, Japan) with the following parameters: tube voltage, 120 kV; tube current, 350 mA; slice thickness, 2.0 mm; pitch factor, 0.938; field of view, 550 mm. The CT images were transferred to the RayStation treatment planning system (v. 10.0.1; RaySearch Laboratories, Stockholm, Sweden). The radiation oncologist contoured the planning target volume (PTV), encompassing the entire left breast, axillary lymph nodes level III, and supraclavicular lymph nodes based on the Radiation Therapy Oncology Group atlas. The VMAT plan was created using two arcs of a 6 MV photon beam with a prescription dose of 50 Gy (D50%) in 25 fractions to the PTV (Figure 1b). The collapsed cone convolution algorithm with a grid size of 0.2 cm was used for dose calculation.

The phantom was placed on the treatment couch and a cone‐beam CT scanner was used to correct the displacement of the phantom setup. Three 2.0 cm × 3.0 cm films were then placed on the phantom surface (the lateral and medial aspects of the left breast and left subclavicular region) as shown in Figure 1c. The left subclavicular region was selected at the flattest possible area of the upper left breast to minimize potential issues with film adherence to curved surfaces. The dose was delivered using a Versa HD linac for eight different thicknesses of topical agents placed on the films.

### 2.5 Statistical analysis

All films were scanned under the same conditions as those used to generate the calibration curve from the ADC value versus the absorbed dose, and the surface doses were estimated using an approximation formula derived from the calibration curve. The samples from the VMAT plan were categorized into two groups based on the topical agent thickness, namely the thin group, which included samples in the range of 0.1–0.5 mm (n= 15), and the thick group, comprising samples with thicknesses of 1.0 and 1.5 mm (n= 6). For each group, mean DER values were calculated by averaging the measurements from all three measurement locations. The Friedman test was conducted using SPSS software (v.27; IBM, NY, USA) to evaluate the null hypothesis, which states that the distributions of StrataXRT, Hirudoid Soft Ointment, and RINDERON‐Vs Ointment are identical. *P*‐values of less than 0.05 were considered statistically significant .

**TABLE 1:** Characteristics of the topical agents.

Brand name	Generic name	Dosage form	Density (g·cm^−3^)	Manufacturer
StrataXRT	Octamethyltrisiloxane	Gel	0.980	Stratpharma
Hirudoid Soft Ointment	Heparinoid	Cream	1.026	Maruho
RINDERON‐Vs Ointment	Betamethasone valerate	Ointment	0.868	Shionogi Pharma

## 3 RESULTS

### 3.1 Conventional irradiation

**FIGURE 2:**
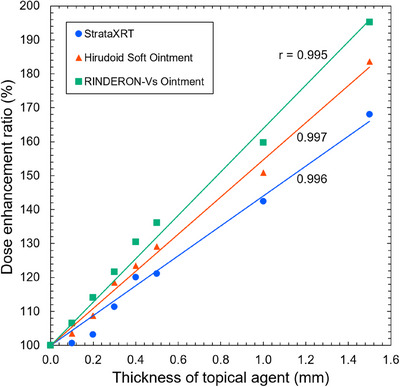
DER for conventional irradiation with varying topical agent thicknesses. DER, dose enhancement ratios.

Table 2 lists the surface doses of the three topical agents measured under conventional irradiation conditions with varying thicknesses. The surface dose increased in the RINDERON‐Vs Ointment, Hirudoid Soft Ointment, and StrataXRT. Figure 2 shows the DER of these agents for different thicknesses. For thicknesses in the range of 0.1–0.5 mm, DER of 101%–121%, 104%–129%, and 107%–136% were observed for StrataXRT, Hirudoid Soft Ointment, and RINDERON‐Vs Ointment, respectively. Furthermore, for thicknesses of 1.0 and 1.5 mm, DER of 142% and 168%, 151% and 184%, and 160% and 195% were observed, respectively.

**TABLE 2:** Surface dose (Gy) for conventional irradiation with varying thicknesses of the topical agents.

Thickness of topical agent (mm)	Surface dose (Gy)		
	StrataXRT	Hirudoid Soft Ointment	RINDERON‐Vs Ointment
0.0	0.62	0.62	0.62
0.1	0.62	0.64	0.66
0.2	0.64	0.67	0.70
0.3	0.69	0.73	0.75
0.4	0.74	0.76	0.81
0.5	0.75	0.80	0.84
1.0	0.88	0.93	0.99
1.5	1.04	1.13	1.21

### 3.2 VMAT irradiation

**FIGURE 3:**
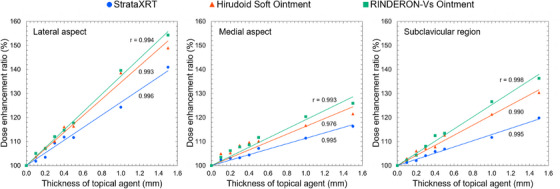
DER for VMAT irradiation at different measurement points with varying thicknesses of the topical agents. DER, dose enhancement ratios; VMAT, volumetric modulated arc therapy.

Table 3 lists the surface doses measured at the medial and lateral aspects of the left breast, as well as the left subclavicular region with varying thicknesses of the three topical agents. At all three measurement points, the surface dose consistently increased in the following order: RINDERON‐Vs Ointment, Hirudoid Soft Ointment, and StrataXRT. Figure 3 shows the DER for these agents for different thicknesses. For thicknesses in the range of 0.1–0.5 mm, DERs of 102%–112%, 104%–116%, and 105%–118% were observed for StrataXRT, Hirudoid Soft Ointment, and RINDERON‐Vs Ointment, respectively, in the lateral aspect of the left breast, 102%–107%, 105%–110%, and 103%–112%, respectively, in the medial aspect of the left breast, and 101%–107%, 103%–113%, and 102%–113%, respectively, in the left subclavicular region. Furthermore, for thicknesses of 1.0 and 1.5 mm, DERs of 124% and 141%, 139% and 149%, and 140% and 154%, respectively, were observed in the lateral aspect of the left breast, 111% and 116%, 117% and 122%, 120% and 126%, respectively, in the medial aspect of the left breast, and 112% and 120%, 121% and 130%, 127% and 136%, respectively, in the left subclavicular region.

**TABLE 3:** Surface dose (Gy) for VMAT irradiation at different regions with varying thicknesses of the topical agents.

Thickness of topical agent (mm)	Lateral aspect Surface dose (Gy)			Medial aspect Surface dose (Gy)			Subclavicular region Surface dose (Gy)		
	StrataXRT	Hirudoid Soft Ointment	RINDERON‐Vs Ointment	StrataXRT	Hirudoid Soft Ointment	RINDERON‐Vs Ointment	StrataXRT	Hirudoid Soft Ointment	RINDERON‐Vs Ointment
0.0	1.24	1.25	1.24	1.56	1.57	1.60	1.33	1.32	1.34
0.1	1.27	1.29	1.31	1.60	1.65	1.65	1.34	1.35	1.37
0.2	1.29	1.34	1.33	1.61	1.66	1.70	1.35	1.39	1.40
0.3	1.36	1.37	1.39	1.61	1.70	1.72	1.38	1.41	1.45
0.4	1.39	1.45	1.43	1.63	1.72	1.74	1.41	1.42	1.51
0.5	1.39	1.45	1.46	1.67	1.73	1.78	1.42	1.48	1.52
1.0	1.55	1.73	1.73	1.74	1.83	1.92	1.48	1.60	1.70
1.5	1.75	1.86	1.92	1.81	1.91	2.01	1.59	1.71	1.83

Abbreviations: VMAT, volumetric modulated arc therapy.

### 3.3 Statistical analysis

**FIGURE 4:**
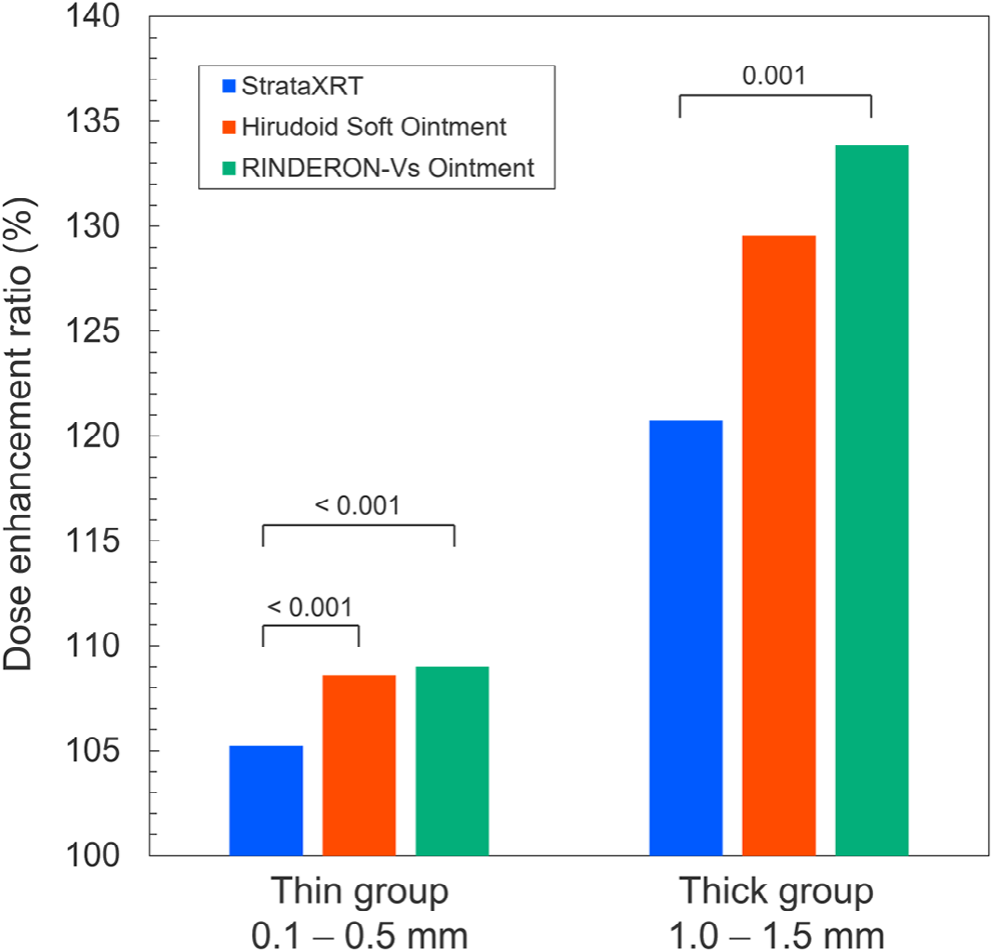
Mean DER values of thin and thick groups. DER, dose enhancement ratios.

Figure 4 shows the DER values of the thin and thick groups. Statistical analysis revealed that StrataXRT yielded a significantly lower DER than the other two agents, whereas there was no significant difference between the Hirudoid Soft Ointment and RINDERON‐Vs Ointment.

## 4 DISCUSSION

This study evaluated the dosimetric effects of StrataXRT on the skin surface in VMAT for breast cancer. Building on previous research on the dosimetric effect of topical agents in VMAT treatment,^18^ this study introduced a novel silicone‐based gel at a new treatment site. When selecting topical agents for radiotherapy, their efficacy and composition must be evaluated, and particular attention should be paid to the potential increase in scatter radiation from high atomic number‐containing products. Despite containing a high atomic number element (Si), StrataXRT did not exhibit a significantly higher DER compared with other topical agents used in this study. Furthermore, other topical agents yielded results that are consistent with those of previous studies.^17^, ^18^ In StrataXRT, we observed mean DER values of 102%–116% at thicknesses of 0.1–0.5 mm, increasing to 116%–126% at 1.0 and 1.5 mm, indicating the lowest DER for StrataXRT among the topical agents evaluated; these results were statistically significant (*p *< 0.05). These findings are consistent with the claims by Stratpharma regarding the bolus effect of StrataXRT.^22^ Notably, this study was conducted independently without the involvement or influence of Stratpharma. The electron density that contributes to the bolus effect depends on the physical density and atomic number. Although the complete composition of StrataXRT has not been fully disclosed, its unique formulation suggests the possibility of a lower effective atomic number than the other topical agents used in this study because of its distinctive composition.

The skin surface dose in breast radiotherapy has undergone significant changes owing to technological advancement. In the early era when physical wedges were utilized, skin doses were high and acute skin reactions posed a substantial challenge. The International Commission on Radiation Units and Measurements recommends that the PTV should be within 95%–107% of the isodose surfaces.^23^ However, it is uncommon to achieve radiation homogeneity in whole breast radiotherapy using a physical wedge. VMAT enhances target dose homogeneity and conformity by delivering multiple segments while rotating continuously around the patient in an arc, thereby mitigating excessive dermal irradiation. In this study, irradiation was performed at a dose of 50 Gy in 25 fractions. Previous research has reported that with fractionated irradiation of 2.0 Gy per day, erythema occurs at total doses of 20–40 Gy, dry desquamation at total doses exceeding 45 Gy, and necrosis at total doses exceeding 60 Gy.^24^ A randomized controlled trial involving patients with breast cancer demonstrated that applying StrataXRT with a thickness of 1.0–2.0 mm, effectively reduced the severity of radiation dermatitis.^25^ This study estimated the total surface dose to be 34–42 Gy in when VMAT technique is used. StrataXRT with thicknesses greater than 0.5 mm may exceed the threshold dose for desquamation and telangiectasia. However, according to the manufacturer's recommendation, StrataXRT should be applied twice daily, and the maximum thickness is expected to be approximately 0.2 mm in clinical settings, which is well below the aforementioned dosimetric threshold. At such thickness, our results demonstrate that the DER remains within the range of 102%–107%. Since topical agents are not visible on treatment planning CT images, the skin dose calculated by the treatment planning system may be underestimated by 2%–7% compared to the actual delivered dose, which warrants consideration in clinical practice.

The methodological limitations of this study include discrepancies between the phantom‐based experiments and actual patient skin conditions. Specifically, the measurement error of Gafchromic EBT4 radiotherapy films (0.9%)^26^, ^27^ and the differences in uniformity between phantoms and actual human bodies^28^, ^29^ must be considered. Moreover, our phantom experiments may not have accurately reproduced the effects of skin movement and deformation on dose distribution during VMAT irradiation. Additionally, while the complete composition of topical agents would be valuable for determining effective attenuation coefficients and theoretical validation, such information is often proprietary and not fully disclosed by manufacturers. This limitation necessitated our empirical approach to dosimetric evaluation. Furthermore, the VMAT conditions used in this study were based on a specific protocol and the results may vary with different dose‐fractionation schemes or field settings.^21^, ^30^, ^31^ Additionally, this study did not address the biological effects of the observed dose enhancement or long‐term patient outcomes.

## 5 CONCLUSION

This paper provides valuable insights into the dosimetric effects of StrataXRT on the skin surface in VMAT for breast cancer, highlighting numerous challenges and future directions. StrataXRT exhibited the lowest DER among the evaluated topical agents in VMAT for breast cancer. However, for thicknesses exceeding 0.5 mm, the dose potentially exceeded the threshold dose for acute skin reactions.

## AUTHOR CONTRIBUTIONS

Tenyoh Suzuki collected the data with Shingo Ohira, performed data analysis, and drafted the manuscript. Shingo Ohira contributed to study design with Mami Ogita and provided overall supervision. Mami Ogita contoured the targets and provided expertise in manuscript preparation. Takeshi Ohta, Yuki Nozawa, Shigeki Saegusa, and Tsuyoshi Ueyama supported experimental procedures. Masanari Minamitani, Takuya Hayashi, Toshikazu Imae, Atsuto Katano, Hideomi Yamashita, Weishan Chang, and Keiichi Nakagawa contributed to data analysis and interpretation, and critically revised the manuscript.

## CONFLICT OF INTEREST STATEMENT

Tenyoh Suzuki, Shingo Ohira, and Keiichi Nakagawa are affiliated with a department that receives unrestricted funding from Elekta AB. However, the funder had no role in this study.
